# A Case of Radiation-Induced Brachial Plexopathy Below the Tolerance Dose

**DOI:** 10.7759/cureus.52283

**Published:** 2024-01-15

**Authors:** Akane Masumoto, Kota Yokoyama, Meika Namba, Kazuma Sasamura, Ryo-ichi Yoshimura

**Affiliations:** 1 Radiation Therapeutics and Oncology, Tokyo Medical and Dental University, Tokyo, JPN; 2 Radiology, Tokyo Medical and Dental University, Tokyo, JPN; 3 Radiology, Japanese Red Cross Musashino Hospital, Tokyo, JPN

**Keywords:** radiation tolerance dose, magnetic resonance imaging, non-small cell lung carcinoma, chemoradiotherapy, radiation-induced brachial plexopathy

## Abstract

This case report details a rare instance of radiation-induced brachial plexopathy (RIBP) occurring below the typical tolerance dose in a 55-year-old woman following chemoradiotherapy for apical non-small cell lung carcinoma. Despite receiving a radiation dose considered safe (47-48 Gray in 25 fractions), she developed sensory abnormalities and motor weakness in the right upper limb. The diagnostic distinction between RIBP and tumor recurrence was achieved using MRI, which showed characteristic features of radiation-induced damage. The patient's medical history included smoking and rheumatoid arthritis, highlighting the role of patient-specific factors in the development of RIBP. The case underscores the importance of recognizing RIBP as a potential diagnosis in patients with new-onset brachial plexopathy post-radiation therapy, even when radiation exposure is within conventional safety limits. This report contributes to the literature by demonstrating that RIBP can occur at lower-than-expected radiation doses, especially in the presence of contributing factors like neurotoxic chemotherapy and individual patient risks. It emphasizes the need for careful assessment and management in such cases to distinguish between RIBP and cancer recurrence.

## Introduction

Radiation-induced brachial plexopathy (RIBP) is a rare delayed nontraumatic complication of radiation therapy for malignancies in the cervical, axilla, and chest wall areas [1]. It was first reported in patients treated with radiation therapy (RT) after surgery for breast cancer [2]. While breast cancer is the most common malignancy leading to RIBP, lung cancer and lymphoma are also notable contributors [1]. A meta-analysis showed that maximum doses to the brachial plexus up to 60-66 Gray (Gy) are generally considered safe, although there are a few reports of RIBP occurring at 41-60 Gy [3]. The estimated three-year incidence of RIBP in patients receiving definitive radiation therapy for apical non-small cell lung carcinoma (NSCLC) was 12%, and only in patients receiving the maximum dose of 78 Gy or more [4]. Herein, we present a rare case of RIBP following combined chemoradiotherapy (CRT) and surgery for apical NSCLC at doses below what is generally considered safe and acceptable.

## Case presentation

**Figure 1:**
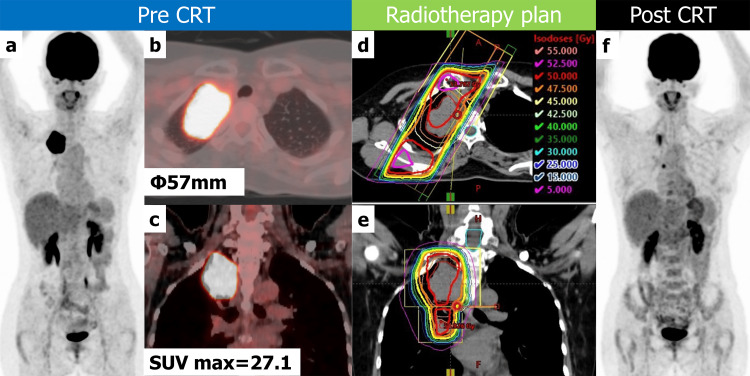
Pre- and post-therapy 18F-FDG PET/CT and treatment planning CT a-c: pre-treatment ^18^F-FDG PET/CT: (a) maximum intensity projection (MIP); (b) axial fusion image of PET/CT with pulmonary window; (c) coronal fusion image of PET/CT with soft tissue window. d-e: radiation planning CT. f: post-treatment ^18^F-FDG PET/CT MIP image. In the pre-treatment positron emission tomography (PET)/CT, there is a 57 mm tumor in the right upper lobe S1 adjacent to the mediastinum, showing an ^18^F-FDG uptake with a maximum standardized value of 27.1 (a-c). In the treatment planning CT, bold circles indicate isodose lines. Thin red, orange, yellow, and cyan circles indicate the gross tumor volume (GTV), clinical target volume (CTV), planning target volume (PTV), and spinal canal, respectively (d, e). In the post-treatment ^18^F-FDG PET/CT, a reduction in the lesion can be observed. The uptake near the vertebrae is non-specific, possibly due to degenerative changes.

A 55-year-old woman with a 34-year history of smoking 20 cigarettes daily had a medical history of rheumatoid arthritis for which she had been temporarily on methotrexate. During a health check, a chest X-ray showed abnormalities. A computed tomography (CT) and subsequent 2-deoxy-2-[^18^F]fluoro-D-glucose positron emission tomography/CT (^18^F-FDG PET/CT) revealed a 5.7 cm tumor in segment S1 of the right upper lobe of the lung, adjacent to the mediastinum without metastasis (Figure 1, a-c). Following a bronchoscopic biopsy, she was diagnosed with squamous cell carcinoma of the right upper lobe lung, staged as cT4N0M0, stage IIIA. For preoperative chemotherapy, she received a first course of cisplatin 80 mg and vinorelbine 50 mg, followed by a second course of cisplatin 60 mg and vinorelbine 40 mg. Concurrently, she underwent preoperative radiotherapy (RT) with 50 Gy over 25 fractions to the right lung tumor and right hilum lymph nodes. RT was delivered with 10 MV photons, using the technique of three-dimensional conformal RT, and the duration of RT was 39 days. During RT, the brachial plexus received a radiation dose of 47-48 Gy (Figure 1, d, e, bold orange circle). One month after completing the CRT, the patient's assessment with an ^18^F-FDG PET/CT scan revealed a decrease in both the size and metabolic activity of the lesion (Figure 1, f). Subsequently, she underwent a right upper lobectomy and a combined parietal pleurectomy.

The patient experienced tingling sensory abnormalities in her right upper limb before and after the surgery, which did not improve despite treatment with pregabalin and duloxetine. Consequently, she was referred to a neurologist eight months post-surgery. Neurological examination revealed decreased pain sensation and abnormal sensation on the ulnar side of the right hand, with nerve conduction velocity and sensory evoked potential tests indicating findings consistent with brachial plexus injury, suggesting a right C8 nerve root injury. No signs of recurrence or metastasis were detected in imaging studies or tumor marker assessments during this period.

**Figure 2:**
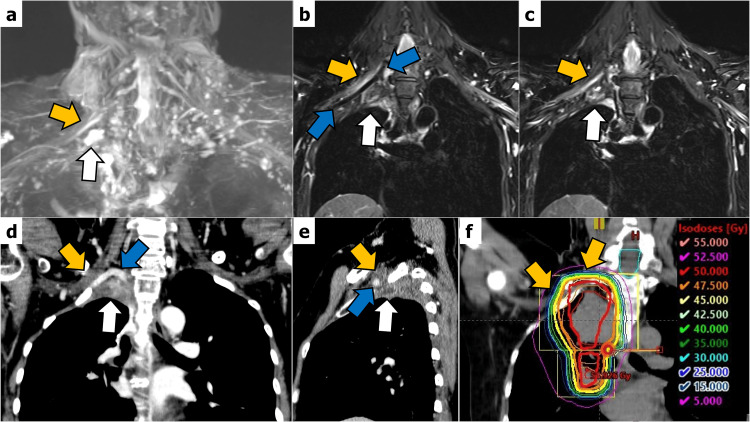
Comparison of postoperative brachial plexus evaluation and radiation planning CT images (a) In three-dimensional (3D) MR neurography, signal enhancement in the right brachial plexus (a, yellow arrow) and discontinuous fluid retention within postoperative scar tissue (a, white arrow) are depicted; (b, c) On the coronal 2D short tau inversion recovery (STIR) images, high signal intensity consistent with the right brachial plexus (yellow arrows) and postoperative scar tissue (white arrows) is observed, with the subclavian artery (blue arrows) intervening; (d, e) On contrast-enhanced CT, the lesion (yellow arrow) and postoperative scar tissue (white arrow) are separated by the right subclavian artery (blue arrows), confirming discontinuity; (f) Compared to the pre-treatment radiation planning CT, the abnormal signal in the brachial plexus corresponds to the area receiving 95% of the prescribed dose of 50 Gy (yellow arrows).

Ten months after the completion of CRT, a cervical spine magnetic resonance imaging (MRI) was performed, revealing localized high signal intensity in the right brachial plexus on both three-dimensional (3D) MR neurography and 2D short tau inversion recovery images (Figure 2 a-c). The contrast-enhanced CT scan showed that the right subclavian artery was running between the area of abnormality seen in the MRI and the post-surgical scar tissue, confirming discontinuity (Figure 2 d, e). When compared to the irradiation field, this corresponded with the area receiving 95 % of the prescribed dose of 50 Gy (Figure 2 f, yellow arrows).

Even though the dose did not reach the generally accepted brachial plexus tolerance threshold of 60-66 Gy, the progression of symptoms and MRI findings, along with the diagnosis of exclusion, suggested the likelihood of radiation-induced neuropathy. It was diagnosed that the patient developed RIBP due to a combination of factors, including surgery and chemotherapy, in addition to radiation exposure.

## Discussion

RIBP is a notable complication that can arise as a delayed consequence of RT for cancers in regions such as the neck, axilla, and chest wall, particularly prevalent in breast cancer treatments [1]. Over the years, the incidence of RIBP has varied, largely influenced by changes in radiation techniques [5]. Previously, high incidence rates of up to 66% were observed due to high radiation doses and different fractionation methods [1,6]. However, with advances in RT, the incidence of RIBP has decreased markedly, with the current incidence below 5% to 0.7% in cases in which the typical plexus dose is kept below 55 Gy [3]. It has been reported that high dose per fraction is a risk factor, and there have been reports of patients with early-stage non-small cell lung cancer receiving stereotactic body radiotherapy developed RIBP with a median maximum dose to the brachial plexus of 30 Gy [7]. Conversely, with normal fractionated irradiation, the likelihood of RIBP occurring below the tolerable dose is extremely low.

The underlying pathophysiology of RIBP is characterized by a complex blend of direct radiation effects leading to initial changes in electrophysiology and histochemistry, followed by fibrosis around the nerve and vascular damage [5]. This progression can result in nerve entrapment and ischemic demyelination. Clinically, the manifestation of RIBP can vary, typically beginning with symptoms like paresthesia and advancing toward severe motor weakness and severe neuropathic pain [8]. Although the progression is generally gradual, rapid worsening of symptoms is also possible in some cases [9]. Neurological symptoms typically emerge between two to four years post-radiation [1] and as early as 1.5 months [10] to several years (up to 20 years) after irradiation [11], correlating with the administered radiation dose. Although a meta-analysis revealed that maximum doses to the brachial plexus up to 60-66 Gy are generally considered safe [3], RIBP can also be influenced by treatment-related and patient-specific factors, indicating that RIBP can occur at radiation doses below established tolerance levels. Treatment-related aspects include the impact of surgical procedures, especially in cases involving hematoma, chronic infections, or extensive lymph node dissections [12]. However, the incidence of perioperative peripheral neuropathy has been reported to be 0.03%, suggesting that direct surgical nerve damage in this case is unlikely [13].

In addition, the use of neurotoxic chemotherapeutic agents such as cisplatin, vinca alkaloids, and taxanes, as well as treatments such as intrathecal methotrexate, are prominent causes. Neuropathy has been reported to occur in 50% to 90% of cases in patients with cumulative cisplatin doses exceeding 500 mg/m2 [14]. Although the cumulative dose of cisplatin in the present case was 140 mg/m2, which is lower than the reported threshold, there have been reports, for example, that nasopharyngeal cancer patients who received cisplatin and concurrent/adjuvant chemoradiation were more likely to develop sensorineural hearing loss than those who received radiation therapy alone [15], It is possible that the combination of radiotherapy and neurotoxic agents such as cisplatin, vinca alkaloids, and taxanes may lower the threshold for each other [5]. In this case, cisplatin and vinorelbine were used, and their combination with radiation therapy may have enhanced the side effects.

On the patient-related side, various factors can predispose individuals to RIBP. These include extremes of age, either very young or elderly patients, and underlying health conditions such as obesity, hypertension, diabetes, and dyslipidemia. Pre-existing peripheral neuropathies, whether due to diabetes, alcohol use, hereditary factors, or other causes, along with arteritis linked to smoking or multiple sclerosis, are also relevant. Moreover, a history of collagen vascular disease and an inherent hypersensitivity in some patients can further elevate the risk of RIBP [12]. The patient had no genetic predisposition, pre-existing peripheral neuropathies, diabetes, or obesity, but she had a history of heavy smoking and rheumatoid arthritis, which may have increased her risk compared to normal subjects.

While there are no direct comparative studies of RIBP that developed below the tolerated dose versus above it, a study of patients who developed RIBP after receiving 65-69 Gy of radiation to the brachial plexus for a pulmonary apex tumor indicated symptoms such as numbness, tingling, and weakness in the shoulder and arm, with a median onset time of 6.5 months [16]. In our patient, who developed RIBP at a lower dose, similar symptoms appeared approximately six months after starting radiation therapy. Although this is based on a limited number of cases, currently, there is no discernible difference in the progression or symptoms between RIBP occurring at low doses and RIBP at high doses. Therefore, it is reasonable to assume that in the present case, a combination of factors is responsible for a condition similar to that of RIBP that develops at doses above the tolerated dose.

The diagnosis of RIBP is challenging, and the process of diagnosing RIBP is centered on distinguishing it from brachial plexopathy caused by tumor infiltration. Needle electroneuromyography plays a pivotal role in this differentiation, showing myokymia in affected patients [17]. Diagnostic imaging techniques such as ultrasound (US) and MRI are crucial for differentiating between RIBP and a recurrence or metastasis of cancer [18]. In the US, RIBP typically manifests as an increased echogenicity, indicating thickening of the brachial plexus and the surrounding fatty tissue. MRI characteristics of RIBP usually include a smooth, longitudinal thickening of the nerves, often accompanied by a T2 hyperintensity and a contrast enhancement [19]. Moreover, perineural fibrosis associated with RIBP can be identified on T1-weighted images as ill-defined tissue that obscures the normal fat planes around the nerves. This fibrosis can appear either hypointense or hyperintense on T2-weighted images, with the hyperintense appearance potentially indicating vascularized scar tissue. On the other hand, tumor involvement or perineural metastasis, while possibly exhibiting similar T1 hypointensity and T2 hyperintensity, tends to present as a nodular and discretely enhancing lesion, which helps in distinguishing it from RIBP [20]. In this case, contrasting the irradiated field with MRI was helpful in the diagnosis. It is very important for the radiation oncologist and the diagnostic radiologist to evaluate and discuss the irradiated field and post-irradiation images when a radiation-induced complication is suspected.

Prevention of RIBP necessitates an accurate comprehension of anatomical structures and imaging-based delineation of organs at risk [21]. This becomes particularly challenging in instances, such as in the present case, where RIBP develops despite radiation doses being within the traditionally accepted safety limits. It is imperative to assess, describe, and meticulously follow up on each patient's unique risk factors for radiation hypersensitivity. The collection and analysis of similar cases are crucial to enhance our understanding of these risks.

In terms of treatment, strategies for RIBP should prioritize symptom alleviation and functional improvement. Neuropathic pain, a frequent and challenging symptom of RIBP, often responds inadequately to conventional analgesics. The reported effectiveness of various medications, including non-opioid analgesics, benzodiazepines, tricyclic antidepressants, and antiepileptic drugs, is notable [5]. The utility of corticosteroids in RIBP management is currently being explored in clinical trials [22]. Hyperbaric oxygen therapy, recognized for increasing oxygenation and promoting tissue recovery, is seen as a potential treatment, but its application requires case-by-case evaluation due to mixed effectiveness reports [23]. In severe cases of RIBP, surgical interventions such as neurotomy are also an option, though their success rates vary [24]. Implementing a multidisciplinary approach, inclusive of non-invasive techniques and physiotherapy, is crucial for effective pain management and limb functionality maintenance.

RIBP can present in various forms. The early transient type typically resolves on its own post-radiation. Conversely, ischemic RIBP, a less common variant, is marked by sudden onset and is often associated with vascular occlusions [3, 9]. This variety underscores the importance of careful monitoring and early intervention, particularly in patients at high risk.

Therefore, it is essential to deepen our understanding of RIBP, ensure early diagnosis, and emphasize the importance of a comprehensive, multidisciplinary evaluation and treatment approach.

## Conclusions

We experienced a case of suspected radiation-induced brachial plexopathy with irradiation below the tolerance dose. Generally, irradiation to the brachial plexus is considered safe up to 60-66 Gy, but there are reports of brachial plexus injuries at lower doses in cases with concomitant cisplatin use, a combination of surgery and chemotherapy, and SBRT where the dose per session is high. Ultimately, care must be taken not to easily attribute the cause solely to radiation effects, and it is important to share information with the attending physician. It is crucial to acknowledge that nerve damage can occur at doses below the tolerance threshold due to multifactorial influences. The correlation between the radiation dose and the brachial plexus, ascertained from treatment planning CT scans and MRI findings, is vital for the early detection of RIBP and its timely management. When RIBP is suspected, comprehensive risk assessment and discussions within a multidisciplinary team are paramount to guarantee effective symptom management and enhance functional outcomes for the patient.
